# Harnessing Soluble NK Cell Killer Receptors for the Generation of Novel Cancer Immune Therapy

**DOI:** 10.1371/journal.pone.0002150

**Published:** 2008-05-14

**Authors:** Tal I. Arnon, Gal Markel, Ahuva Bar-Ilan, Jacob Hanna, Eyal Fima, Fabrice Benchetrit, Ruth Galili, Adelheid Cerwenka, Daniel Benharroch, Netta Sion-Vardy, Angel Porgador, Ofer Mandelboim

**Affiliations:** 1 Division of Biology, California Institute of Technology, Pasadena, California, United States of America; 2 Sheba Cancer Research Center, Sheba Medical Center, Tel Hashomer, Israel; 3 The Shraga Segal Department of Microbiology and Immunology and the National Institute for Biotechnology in the Negev, Ben Gurion University of the Negev, Beer Sheva, Israel; 4 The Whitehead Institute for Biomedical Research, Cambridge, Massachusetts, United States of America; 5 Lautenberg Center for General and Tumor Immunology, The Hebrew University Hadassah Medical School, Jerusalem, Israel; 6 Division of Innate Immunity, German Cancer Research Center, Heidelberg, Germany; 7 The Pathology Department, Soroka Hospital, Beer Sheva, Israel; Beijing Institute of Infectious Diseases, China

## Abstract

The natural cytotoxic receptors (NCRs) are a unique set of activating proteins expressed mainly on the surface of natural killer (NK) cells. The NCRs, which include three members; NKp46, NKp44 and NKp30, are critically involved in NK cytotoxicity against different targets, including a wide range of tumor cells derived from various origins. Even though the tumor ligands of the NCRs have not been identified yet, the selective manner by which these receptors target tumor cells may provide an excellent basis for the development of novel anti-tumor therapies. To test the potential use of the NCRs as anti-tumor agents, we generated soluble NCR-Ig fusion proteins in which the constant region of human IgG1 was fused to the extracellular portion of the receptor. We demonstrate, using two different human prostate cancer cell lines, that treatment with NKp30-Ig, dramatically inhibits tumor growth *in vivo*. Activated macrophages were shown to mediate an ADCC response against the NKp30-Ig coated prostate cell lines. Finally, the Ig fusion proteins were also demonstrated to discriminate between benign prostate hyperplasia and prostate cancer. This may provide a novel diagnostic modality in the difficult task of differentiating between these highly common pathological conditions.

## Introduction

Immune mechanisms are thought to provide essential protection from the development of cancer diseases. Studies of human patients and mice models have shown that deficiencies in key immunologic components lead to increased susceptibility to the development of cancer [1]–[3]. Natural killer (NK) cells are an important subset of cytotoxic lymphocytes that belong to the innate immune response and are best characterized by their ability to spontaneously kill virally infected and tumor cells [4]. The efficiency by which NK cells destroy a wide range of cell lines suggests an important role in immunosurveillance. Indeed, accumulating clinical and experimental data demonstrate the importance of NK activity in cancer elimination *in vivo*; in mice, depletion of NK cells results in a significantly increased susceptibility to chemically induced cancer [1], while in leukemia patients reduced NK cytotoxicity was shown to strictly correlate with enhanced progression of the disease [5].

The activation of NK cells is regulated by a set of surface receptors that either induce or inhibit the cytotoxic response [4], [6]. The main mechanism that controls NK inhibition is based on the recognition of MHC class I molecules by NK inhibitory receptors, such as the killer-Ig-like receptors (KIRs) and the CD94/NKG2A complex. This mechanism ensures that the NK cells are continuously inhibited from killing healthy cells that express normal levels of MHC class I proteins [7]. However, while MHC class I expression is essential in order to inhibit NK cytotoxicity, down-regulation is not enough to induce a response, as specific activation signals are also required. These signals are delivered by a set of lysis receptors that recognize non-MHC class I ligands, expressed on target cells [8]. Thus, NK cells are not only capable of sensing the absence of MHC class I proteins, but are also equipped with surface receptors that allow specific detection of their targets.

The main NK activating receptors involved in recognition and killing of tumors include the NKG2D homodimer and the three natural cytotoxic receptors (NCRs) NKp46, NKp44 and NKp30 [8]. The relative contribution of each of these receptors to NK cytotoxicity against tumors differs, indicating the existence of various specific lysis ligands [9]. Indeed, several stress inducible cellular proteins have been identified as ligands for the NKG2D receptor: the MHC class I chain related antigens (MICA and MICB) and the UL16 binding proteins (ULBP1-4) [10].

In contrast to the NKG2D, the cellular ligands of the NCRs are currently unknown and the only NCRs ligands identified so far are virally derived proteins [11]–[14]. However, substantial evidence indicates that cellular ligands for the NCRs do exist and that their expression is critical for the ability of NK cells to destroy tumors [9], [15]–[17]. These findings are supported by clinical studies showing for several cases of AML a correlation between low levels of NCRs ligands in AML patients and a poor prognosis of the disease [18]. Furthermore, we have recently shown that deletion of a single NCR gene, the NKp46 mouse homologue (NCR1), significantly reduces the ability of NK cells to clear tumor cells *in vivo* [19].

In the past decade, cancer immunotherapy studies have extensively focused on the attempt to exploit the highly specific nature of the adaptive immune response for the development of novel treatments. This approach led to the generation of several humanized antibodies directed against specific tumor antigens. The impressive clinical success of the antibody-based therapy opened the gate for an intensive search for additional highly specific tumor markers and since 1995 several antibodies have been approved for clinical use while more are currently being evaluated in oncology trials [20], [21].

To expand this method, various tools have been applied in an attempt to identify new tumor antigens that would be suitable for a wider range of cancers and still retain selective recognition. The NCRs represent a unique example for proteins that have acquired such a broad specificity and are highly adapted to recognize cancer cells. To translate this potential into a therapeutic tool, we have generated immunoglobulin (Ig) fusion proteins that contain the extracellular portion of the receptor fused to the constant region of human IgG1. Thus, similar to the antibody-based tumor therapy approach, we hypothesized the NCR-Ig fusion proteins may be used as ‘targeted missiles’; the extracellular portion provides tumor specificity while the constant region of the human IgG1 (Fc) allows recruitment of immune components, which enhance specific tumor elimination. Here we show the potential use of such therapy using human prostate cancers models.

## Materials and Methods

### Cells

We used the human prostate cancer cell line DU145 and the human prostate cancer cell line PC3/*Luc*, which was prepared as described before [14]. Briefly, PC3.38, a clone of the human prostate adenocarcinoma cell line, derived from PC3 (ATCC, CRL-1435), was infected with recombinant rLNC/*Luc* retrovirus (expressing luciferase gene downstream to CMV promoter) and selected by G418 (400 µg/ml) generating PC3/*Luc* clone. Stable expression of luciferase in cell culture was routinely confirmed using the CCCD camera.

### Fusion proteins and flow cytometry analysis

The production of NKp30-Ig, NKp46D2-Ig, NKp46D1-Ig and CD99-Ig was described before [13]. Control human IgG1 protein (hIgG1 kappa, PHP010) was purchased from Serotec (Oxford, UK).

### Immunohistochemistry

Prostatic tissues, both hyperplasic and malignant, were fixed in buffered formalin. Microwave heating of the formalin-fixed, paraffin embedded tissue sections in citrate buffer was performed to retrieve antigens. Sections were then stained by the different NCR-Igs or control-Ig (8 µg/ml, final concentration) followed by biotinylated-goat-anti-human-Fc (Jackson ImmunoResearch, West Grove, PA). For detection, the avidin-biotin peroxidase complex method was employed with Vectastain kit (Vector Laboratories, Burlingame, CA). Staining was graded as the percentage of positive prostatic cells (hyperplasic or malignant) from a total of 100 cells. We also evaluated the intensity of the staining as 0 = non, 1 = weak, 2 = moderate and 3 = strong. Stained sections were analyzed by two pathologists. Immunohistochemical results were considered as positive whenever the percentage of positively stained prostatic cells surpassed 50% and the intensity was higher than 1.

### Tumor implantation and treatment

For the DU145 model we s.c. injected male nude mice with the human prostate tumor line DU145 (4×10^6^). Two weeks after injection when the tumors became visible, mice were treated with NKp30-Ig, NKp46D2-Ig, control human IgG1 or PBS. Treatments were administered 5 times (days 2, 7, 15, 25 and 34) and included an initial larger dose of the proteins (20mg/kg) followed by lower doses (10mg/kg). Tumor progression was evaluated every three days by measuring the diameter of the tumors.

For the PC3/*Luc* model, male nude mice (4–8 weeks old) were injected with 15×10^6^ PC3/*Luc* cells into the s.c. space of the left flank of each mouse. Three weeks after tumor implantation, mice were injected i.p. with 4 µg/kg of NKp30-Ig, NKp46D2-Ig, control human IgG1 or PBS every other day over a period of 3-4 weeks.

### Imaging of tumor PC3/*Luc* xenografts

All mice were monitored for tumor expression before and at the end of the treatment procedures. Only those mice that within 21 days from injection expressed a detectable size of tumor, as evaluated by the CCCD camera, were chosen for further manipulations. Before imaging, mice were anesthetized with 4% chloral hydrate (Fluka-Sigma, Israel). Five minutes prior to imaging, mice were injected i.p. with 126mg/kg body weight of aqueous solution of Beetle lucifering (Promega Corp., Madison, WI). The mice were then placed in a light-tight chamber of a CCCD camera system (Roper Scientific, Princeton Instrument, Trenton NJ) and photographed first with a supplemented controlled light in order to take a gray-scale body reference image. Photons emitted from the mouse were then detected in complete darkness, collected and integrated for a period of 2 min. A pseudo color image represents light intensity (blue as the least intense and red as the most intense). Measurements are an integration of the total sum of signals detected, subtracted by the background integrated light emitted from an equal area in the same mouse.

### Pharmacokinetic analysis *in vivo*

Female CB17.SCID mice were injected i.p. with a single dose (5mg/kg body weight) of NKp46D2-Ig or NKp30-Ig. Levels of fusion proteins in the serum were determined at different time points, including 0 (pre-dose) 0.25, 0.5, 1, 2, 6, 24, 48, 96, 168, 216, 264 and 336 hours post dose-administration. At each time point, 3 mice were sacrificed blood samples were collected into separation tubes, which were incubated at room temperature for 30 minutes prior to centrifugation (to allow clotting). After 30 minutes, the tubes were immediately centrifuged (10 minutes at 10,000rpm at room temperature) and serum was collected. Levels of NKp30-Ig or NKp46D2-Ig fusion proteins in the serum were analyzed using a standard ELISA assay.

### Apoptosis assay

PC3/*Luc* or DU145 cells (50,000/ml) were incubated with 5, 10 or 50 µg/ml of NKp30-Ig, NKp46D2-Ig, NKp46D1-Ig or PBS for 2 hours on ice. Cross -linking antibody (against human IgG1) was than added at final concentrations that equal to 1/10 of the amount of fusion protein added previously (0.5, 1 or 5 µg/ml). Cells were than incubated at 37°C for 48 hours and the percentage of apoptotic cells was determined using a standard Annexin V/PI analysis.

### Macrophage-mediated killing assays

As effector cells we used peritoneal macrophages derived from CD1 nude mice 5 days after Thioglycolate injection. Retrieved macrophages were than activated for 2h with LPS (1mg/ml). Radioactive labeled PC3/*Luc* or DU145 cells (1*10^4^/well at flat 96-well plate) were incubated with the LPS-activated macrophages at the indicated E∶T ratios. Specific lysis was determined after 48 hours.

## Results

### Recognition of malignant primary human prostate cancer by NKp30 and NKp46

NKp46 and NKp30 are constrictively expressed on the surface of resting as well as activated NK cells and are dominantly involved in the killing of various tumor cell lines *in vitro*. However, since the cellular ligands of these receptors are still unknown, little is known about their expression pattern and distribution in different pathological settings.

**Figure 1:**
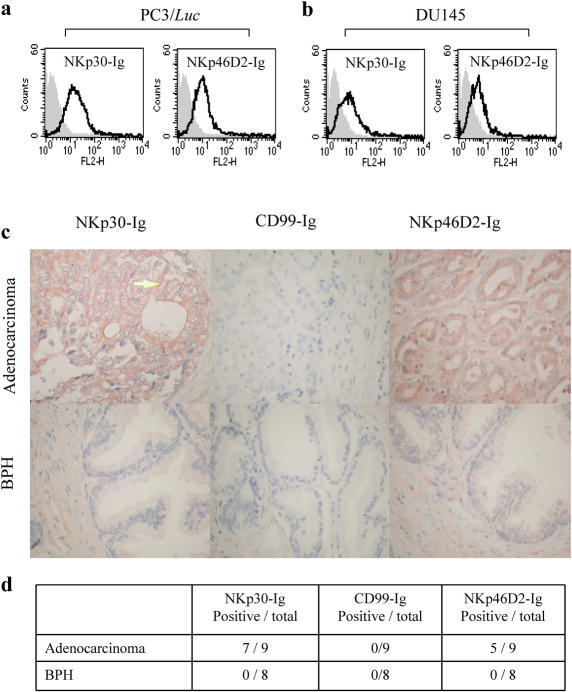
Expression of NCRs ligands on human prostate cancer. (a,b) NKp30-Ig and NKp46D2-Ig specifically bind to human prostate cell lines. PC3/*Luc* (a) and DU145 (b) cell lines were stained with NKp30-Ig, NKp46D2-Ig or control CD99-Ig, followed by PE-conjugated mouse anti-human IgG1 antibody. Grey histograms represent the background staining by the control CD99-Ig fusion protein and the black empty histograms represent the staining by either NKp30-Ig or NKp46D2-Ig, as indicated in the top of each histogram. This figure represents one experiment out of three performed. (c) Immunohistochemical staining of primary human prostate adenocarcinoma and benign prostate hyperplasia (BPH) by NKp30-Ig and NKp46D2-Ig. Cuts from formalin-fixed and paraffin-embedded human prostate adenocarcinoma (upper panel) and BPH (lower panel) were antigen-retrieved by microwave-citrate treatment. Slides were then stained with NKp30-Ig, negative control CD99-Ig or NKp46D2-Ig, followed by biotinylated-goat-anti-human-Fc and avidin-biotin HRP complex. Substrate for HRP was AEC (red color) and slides were counter-stained with Hematoxylin. Figure shows a representative staining at X400 magnification. Arrow in NKp30-Ig staining of adenocarcinoma (top left panel) points to a representative membrane staining. Staining intensity for top left and top right panels is considered as 2 (see Methods). (d) Expression of NKp30 and NKp46 ligands is abundant on malignant prostate tumors. Cuts from different patients suffering from benign (*n* = 8) or malignant (*n* = 9) prostate tumors were prepared and stained as above. Staining was performed in triplicates. Analysis of staining intensity (0-3) and percentage of stained tumor cells was performed by two pathologists. Positive staining was defined when staining intensity was above 1 and encompassed at least 50% of the cells, as described in ‘material and methods’.

Prostate cancer is the most frequently diagnosed solid tumor among men in the United States and the second leading cause of cancer deaths in western countries [22]. Unfortunately, there are no effective therapies available today for the fatal hormone-refractory stage of the disease. To test whether human prostate tumors are recognized by the NCRs we stained the PC3/*Luc* and DU145 cell lines with NKp30 and NKp46 proteins fused to human IgG1. We have previously shown that the binding site of NKp46 to various tumors is located at the membrane proximal domain and the steam region of the receptor (D2) and that the expression of D2 fused to human IgG (NKp46D2-Ig) enables better recognition of target cells [13]. As shown in figure 1a and b, both PC3/*Luc* and DU145 cells were specifically stained by NKp30-Ig and NKp46D2-Ig, but not by the control CD99-Ig (grey histograms), thus indicating the expression of ligands for these two NK activating receptors on prostate tumor cell lines.

To extenuate our observations, we analyzed the expression of ligands to NKp30 and NKp46 on primary prostatic adenocarcinoma and benign prostatic hyperplasia (BPH) derived from human patients. Formalin-fixed paraffin-embedded prostatic cancers were stained with NKp30-Ig, NKp46D2-Ig or the control fusion proteins (such as CD99-Ig). Figure 1c shows representative examples of stained tissues and figure 1d summarizes the results obtained from 9 adenocarcinoma and 8 benign prostate cancer tissues. As shown, 7/9 and 5/9 prostate adenocarcinomas were positively stained by NKp30-Ig and NKp46D2-Ig, respectively, indicating the existence of ligands for NKp30 and NKp46 on the malignant cells. The expression of these antigens encompassed 50–95% of tumor cells with different degrees of intensities and showed both intracellular and membranal distribution (Figure 1c, arrow at the top left panel points to membrane staining). In contrast, all BPH sections tested were negatively stained by NKp30-Ig or NKp46D2-Ig, as in most cases less than 10% of the cells were stained and the intensity was bellow 1. Importantly, neither the adenocarcinomas nor the BPH sections were stained by the control Ig fusion protein CD99-Ig (and other control proteins, data not shown). These results demonstrate that similarly to prostate cancer cell lines grown in culture, primary derived tumors selectively express ligands for the NK lysis receptors NKp30 and NKp46D2. Furthermore, the expression of these unknown ligands is induced only at later stages of the disease in which adenocarcinoma has already evolved.

### NKp30-Ig inhibits the growth of the prostate cancer cell line DU145 *in vivo*

Ig fusion proteins have been previously utilized as effective therapeutic agents in clinical practice [23]. Here we demonstrate that both NKp30-Ig and NKp46D2-Ig specifically bind human tissues derived from prostate cancer patients, indicating the presence of specific (although unknown) ligands (figure 1b and c).

**Figure 2:**
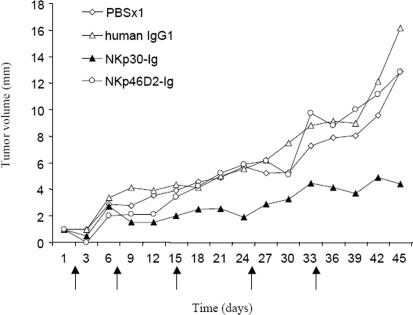
NKp30-Ig treatment reduces growth of prostate cell line DU145 *in vivo*. Male nude mice injected with the human prostate tumor line DU145 (4×10^6^), were treated with NKp30-Ig (*n* = 10), NKp46D2-Ig (*n* = 9), control human IgG1 (*n* = 10), or PBS (*n* = 10). Day 1 is defined at two weeks post-injection when tumors became visible. Treatments were administered 5 times (days 1, 7, 15, 25 and 34, marked by arrows) and included an initial larger dose of the proteins (20 mg/kg) followed by lower doses (10 mg/kg). Tumor progression was evaluated every three days by measuring the diameter of the tumors. The graphs show the average diameter (mm) of the tumors in each group measured in days 1–45.

To determine whether NKp30 and NKp46D2 fused to human IgG1 could mediate effective anti tumor response *in vivo*, we injected nude mice with the human prostate tumor line DU145. Two weeks after injection, when the tumors became visible, (defined as day 1 in figure 2) mice were treated with NKp30-Ig, NKp46D2-Ig, control human IgG1 or PBS (note legend to figure 2 for treatment course and growth evaluation). In both control groups receiving human IgG1 (*n* = 10) or PBS (*n* = 10), animals demonstrated a rapid growth of tumors (figure 2). Similarly, the NKp46D2-Ig treated mice (*n* = 9) showed a gradual increase in tumors size, indicating no therapeutic effect. In contrast, treatment with NKp30-Ig (*n* = 10) resulted in a remarkable suppression of tumor development; as from the second administration of NKp30-Ig (at day 7), tumors size remained constant for three weeks and only a moderate progression was detected in the following days (figure 2). Furthermore, in 5 out of 10 mice that were injected with NKp30-Ig, the tumors disappeared after the second injection (day 7) and did not reappear even up to two months after the last injection.

### Treatment with NKp30-Ig reduces PC3/*Luc* tumor growth *in-vivo*

Studying a complex biological process such as tumor growth and therapy impact *in-vivo* requires a model that is both sensitive and accurate enough to detect even subtle developments. Recently, a new bioluminescence based imaging technology that allows non-invasive detection of tumor cells *in-vivo* was reported [14], [24]. This strategy relies on the engraftment of human cancers that stably express a reporter gene, such as *luciferase* (*Luc*), into mice. Expression of luciferase enables *in-vivo* monitoring of the relative size and distribution of the tumors and can therefore also detect metastatic development. Furthermore, this system is highly sensitive and reliable in detection of minor tumors that are difficult or even impossible to sense in other methods such as direct tumor size measurements or FACS analysis of tumor extracts [24]. For this reason, we used the human prostate cancer cell line PC3 labeled by stable expression of luciferase gene (PC3/*luc*) that was previously applied in other similar *in-vivo* models [14].

To monitor the effect of NKp30-Ig and NKp46D2-Ig fusion proteins on the progression of PC3/*luc* prostate cancer cell line *in-vivo*, we injected male nude mice with PC3/*luc* cells. Three weeks after injection (designated as ‘start point’), tumor growth was assessed by the CCCD camera and those mice expressing tumors that were large enough for transmitting an integrated signal intensity of 100 photon counts and above, were chosen for further treatment with NKp30-Ig (*n* = 16), NKp46D2-Ig (*n* = 9) or with PBS as control (*n* = 8). The treatment included an i.p. injection of PBS or 4mg/kg body weight of the relevant fusion protein, given every other day for a month. In the end of the treatment period (designated as ‘end point’), tumor size was evaluated again in the CCCD camera.

**Figure 3 pone-0002150-g003:**
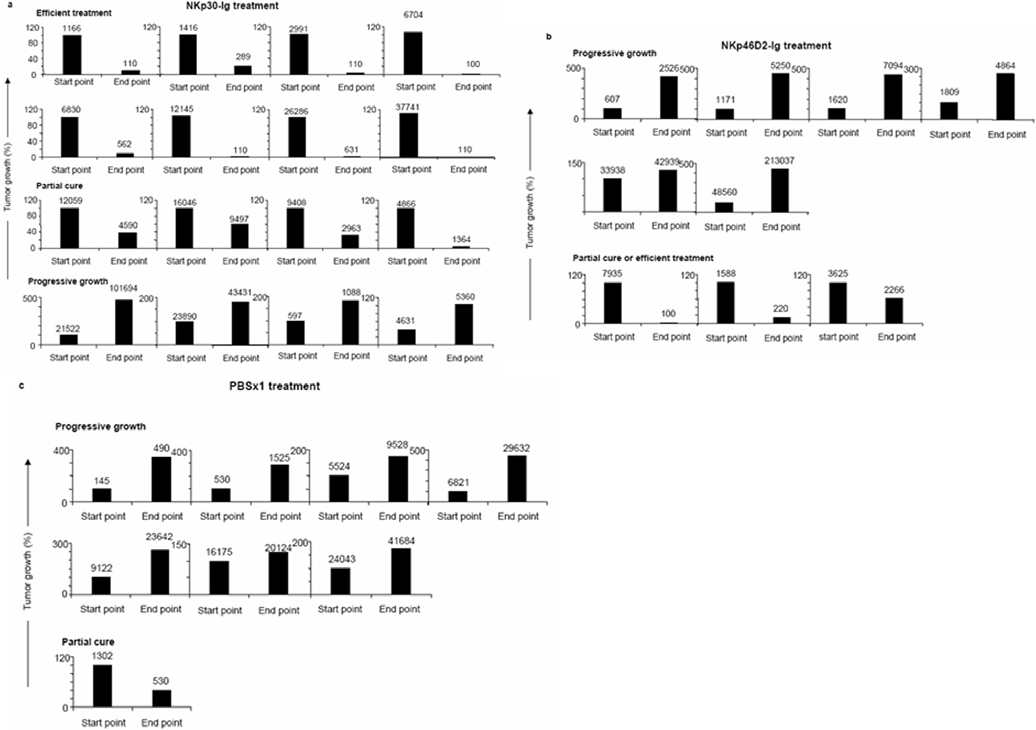
Treatment with NKp30-Ig reduces PC3/*Luc* tumor growth. Male nude mice were injected with PC3/*luc* tumor cells (15×10^6^) into the SC left flanks. Three weeks after tumor implantation, mice were injected (i.p.) every second day over a period of one month with 4mg/kg of NKp30-Ig (*n* = 16) (a), NKp46D2-Ig (*n* = 9) (b) or PBS (*n* = 8) (c). Tumor progression was monitored by measuring light emission from each individual mouse in the initiation (‘start point’) and in the end (‘end point’) of the treatment period. Y-axes represent the relative (in percentage) changes in tumor size after treatment, as calculated from the integrated light emission measured in each time point (indicated numbers above columns). Shrinkage of the tumor by 20% or below its original size was referred as ‘efficient treatment’. This figure is a summary of two experiments and includes all the mice that were tested.

**Figure 4:**
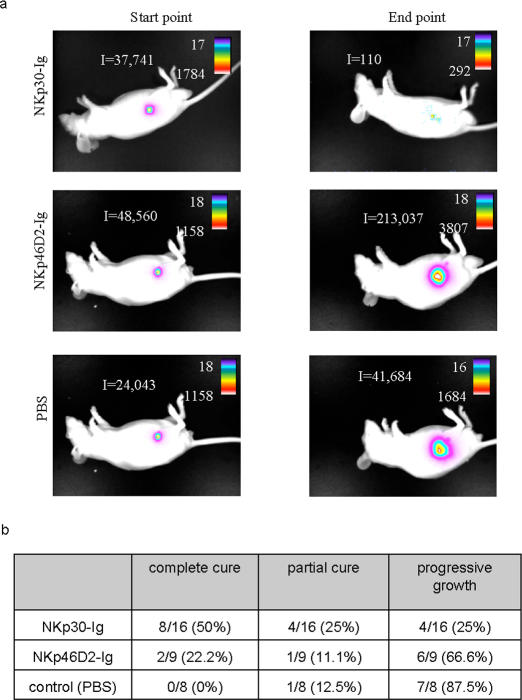
Summary of fusion protein treatment. (a) Visualization of tumor progression and distribution *in vivo*. The figure shows an image visualization of one representative animal of each treatment. The scale on the right of each figure describes the color map of the photon count. The integrated light emission (‘I’) is indicated in the left of each photo. (b) Summary of treatment effect. Table describes the overall effect of treatments, as shown in details in figure 3.

As shown in figure 3a and summarized in figure 4b, treatment of mice with NKp30-Ig had a dramatic effect on tumor growth. While in the control group (figure 3c and 4), rapid increase in tumor size was observed in almost all the animals (87.5%), NKp30-Ig-treatement led to a substantial reduction in tumor progression; in 50% of the mice treated with NKp30-Ig the tumor was drastically reduced to 20% or bellow of its original size (regarded as ‘efficient treatment’), 25% showed partial effect while 25% did not respond and developed progressive tumors. Moreover, in those mice in which efficient treatment was obtained, no relapse was observed even 3 month after the last NKp30-Ig administration. Importantly, NKp30-Ig therapeutic effect was not dependent on the original size of the tumor, as responsiveness or unresponsiveness did not correlate with the initial signal detected from the tumor, as measured in the ‘start point’ (indicated in numbers above each column).

NKp30-Ig treatment significantly suppressed growth of both DU145 and PC3/*Luc*, yet with a more prominent effect on PC3/*Luc* (figures 2–4). This could not be attributed to differences in ligand expression (figure 1a) and could reflect the enhanced progressive growth of DU145 compared to PC3/*Luc*. In contrast to NKp30-Ig and in agreement with the results presented above (figure 2), NKp46D2-Ig treatment had only a marginal effect (figure 3b and 4); 66.6% of the mice treated with NKp46D2-Ig showed progressive tumor growth, while 22.2% and 11.1% were partially cured or effectively treated, respectively.

Figure 4a shows an illustration of one representative mouse from each treatment group. In most cases no metastases were observed. The tumor measurements described above (figure 3) represent the entire tumor mass detected in each animal.

These results clearly demonstrate the therapeutic potential of NKp30-Ig fusion protein for human prostate cancer treatment *in vivo*.

### Pharmacokinetics of NKp30-Ig and NKp46D2-Ig

Taken together, our results indicate that NKp30-Ig, but not NKp46D2-Ig, can suppress tumor growth *in vivo*. This selective effect was surprising since both NKp30-Ig and NKp46D2-Ig similarly bind to PC3/*Luc*, DU145 (figure 1a and b) and human prostate cancer specimens *in vitro* (figure 1c). Thus, the differences between the therapeutic potential of NKp46D2-Ig and NKp30-Ig must result from other characteristics of the proteins that influence the efficiency of treatment.

One of the most important factors in cancer therapy is the t_1/2_ of the therapeutic agent. In order to mediate a significant anti-tumor effect the fusion proteins must be able to reach the serum and remain stable for several hours.

**Figure 5:**
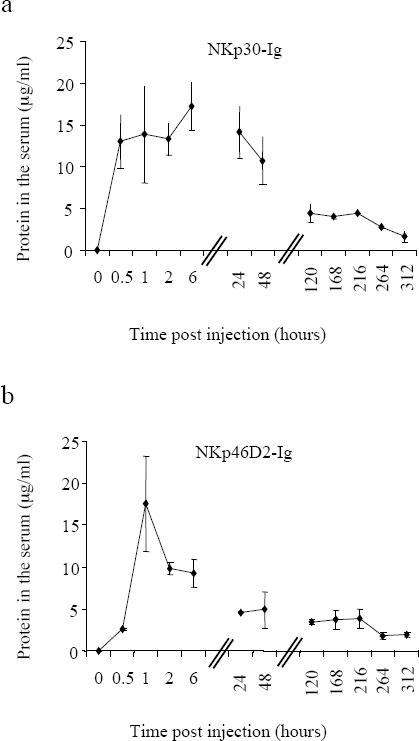
Pharmacokinetics of NKp30-Ig and NKp46D2-Ig fusion proteins *in vivo.* Mice were injected i.p. with one dose (5mg/kg) of NKp30-Ig (a) or NKp46D2-Ig (b). Serum sample were collected (at 0, 0.5, 1, 2, 6, 25, 48, 120, 168, 264 and 312 hours after injection) and levels of NKp30-Ig or NKp46D2-Ig were determined in a standard ELISA assay. Figure shows the average amount of fusion proteins detected in the serum of three mice, measure at each time point. Error bars represent mean±s.d of triplicates.

To estimate the relative stability of the fusion proteins *in vivo*, mice were given a single dose (5mg/kg body weight) of either NKp30-Ig or NKp46D2-Ig and then monitored for specific protein levels in the serum every few hours for 14 days. The maximal level of proteins measured in the serum was similar for both NKp46D2-Ig and NKp30-Ig (figure 5a and b). However, clear differences were observed in the kinetics and stability of the two fusion proteins; high levels of NKp46D2-Ig were identified in the serum after 1 hour from injection, albeit decreased rapidly in the blood and within 2 hours almost 50% of the protein was degraded. Moreover, after 24 hours, only small traces of NKp46D2-Ig were found (figure 5b). In contrast, highest levels of NKp30-Ig were detected in the serum already 30 minutes after injection and were maintained for almost 2 days, decreasing only after 120 hours (figure 1a). These observations demonstrate the relative stability of NKp30-Ig *in vivo* and may explain, at least partially, the failure of NKp46D2-Ig to produce a therapeutic effect.

### NKp30-Ig enhances macrophages-mediated cytotoxicity against tumor cells *in vitro*

**Figure 6:**
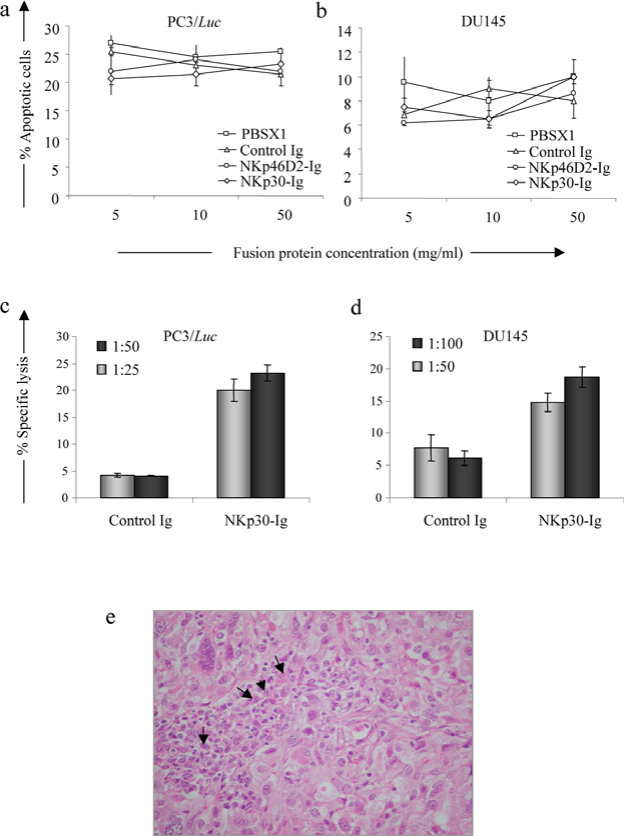
Mechanism of NKp30-Ig mediated tumor regression. (a,b) NKp30-Ig does not induce apoptosis in tumor cells. PC3/*luc* (a) or DU145 (b) cells were incubated with increasing concentrations of NKp30-Ig, NKp46D2-Ig, control Ig or PBS in the presence of a cross-linking antibody. After 48 hours, the percentage of apoptotic cells was determined by Annexin V and PI staining. The figure shows one out of three experiments performed. (c,d) NKp30-Ig can mediate tumor opsonization by macrophages. Radioactive labeled PC3/*Luc* (c) or DU145 (d) cells were incubated with LPS-activated macrophages at the indicated E∶T ratios. Specific lysis was determined after 48 hours. Error bars represent mean±s.d of triplicates. Figure represents one out of three experiments performed. (e) Infiltration of macrophages to the tumor tissue. Human prostate tumors (DU145) grown in nude mice were fixed in 10% buffered formalin. Paraffin-embedded sections were stained in Hematoxylin and Eosin. The arrows indicate tumor associated- macrophages (X380). This figure represents one out of 5 sections tested.

Several mechanisms have been proposed for the ability of tumor antibodies to mediate their effect *in vivo*. One possible way by which such antibodies can inhibit tumor growth is by binding to surface receptors that are involved in cell cycle regulation [25]. We therefore first tested whether binding of NKp30-Ig to its unknown tumor ligands can induce direct apoptosis or growth arrest of tumor cells in culture. PC3/*luc* and DU145 cells were incubated with increasing concentrations of NKp30-Ig, NKp46D2-Ig or control Ig fusion protein for 48 hours in the presence of a cross linking antibody. Following treatment, the percentage of apoptotic cells was determined by Annexin V and propidium iodide (PI) staining. Incubation of either PC3/*luc* or DU145 cells with the various fusion proteins had no effect on the apoptosis rates (figure 6a and b) as the mean spontaneous apoptosis of PC3/*luc* and DU145 cells (around 25% and 8%, respectively) remained similar and was not affected by any of the treatments. In addition, no cell cycle arrest was observed, as evaluated by thymidine incorporation assays (data not shown). Similar results were obtained following incubation for 24 h or 72 h (data not shown).

Since the tumor cells did not demonstrate any intrinsic sensitivity to NKp30-Ig *in vitro*, we next tested the capacity of NKp30-Ig to induce effector-mediated killing of tumor cells. Macrophages have been previously implicated as major players in antibody-dependent tumor control *in vivo* [26], [27]. To evaluate the ability of NKp30-Ig to enhance macrophages-mediated lysis of tumor cells, mice were first injected (i.p.) with thioglycolate in order to cause a local non-pathogenic inflammation and recruit large numbers of macrophages into the peritoneum cavity. Five days later, mice were sacrificed and macrophages were isolated. To enhance the cytolytic activity, macrophages were activated in culture with LPS and than tested for the ability to lyse PC3/*luc* and DU145 cells coated with NKp30-Ig or control Ig fusion protein, at various E∶T ratios. Little or no killing was observed when cells were coated with a control Ig fusion protein, even at the highest E∶T ratios tested (50∶1 and 100∶1 for PC3/*luc* and DU145 cells, respectively, figure 6c and d). In contrast, pre-incubation of both PC3/*luc* and DU145 cells with NKp30-Ig resulted in an enhanced lysis of the targets. It should be noted that, although higher E∶T rations were required in order to efficiently lyse the DU145 cells, as compared with PC3/*luc* cells, both targets were sensitive to NKp30-Ig mediated killing by activated macrophages. We did not observe any effect of NKp30-Ig on the ability of freshly isolated NK cells or complement components to kill PC3/*luc* and DU145 cells *in vitro* (data not shown). Finally, histological examination of the DU145 tumor tissue revealed filtrates of immune cells including macrophages (Fig. 6c, pointed by black arrows), further indicating a possible involvement of macrophages in the clearance of prostate xenographts. Thus, the inhibition of tumor growth *in vivo* by NKp30-Ig may depend, at least partially, on a macrophages mediated response.

## Discussion

A key factor in effective therapy is the specificity of the therapeutic agent. Cancer immunotherapy attempts to exploit the highly specific nature of the immune system to treat malignancy by using humanized monoclonal antibodies or Ig fusion proteins that selectively bind to tumor antigens. However, finding a suitable antigen is a critical step that has often proved to be difficult, especially in the case of solid tumors. Importantly, while the adaptive immune response successfully produces tumor specific antibodies and CTLs that may be harnessed for clinical use, these are often limited to a specific type of cancer and are therefore suitable for only a minor fraction of the patients. Thus, the identification of tumor specific markers with a broader specificity is of great importance.

Since innate immune mechanisms are evolutionary designed to respond to a broad spectrum of pathological conditions, including cancer, applying the molecular mechanisms that mediate this recognition may help to develop new anti-tumor medicines. The natural cytotoxic receptors (NCRs), which are expressed on NK cells, are an attractive example of such proteins with specificity for tumor ligands commonly expressed on multiple types of transformed cells. In light of the restricted fashion by which these ligands are primarily confined to tumors, these molecules may represent an excellent target for cancer therapy. We published that tumor-associated heparan sulfate molecules are involved as co-ligands for NCRs [28]–[31]. However, since the identity of the heparan sulfate epitopes and of the other NCRs' ligands is still unknown, direct targeting is impossible. In this study we suggest an alternative approach; by generating NCR-Ig fusion proteins we allow the NK receptors to bind to their preferred (and mostly unknown) tumor ligands and to selectively recruit, via the Ig domain, effector mechanisms against the malignant cells. Whether NKp30-Ig therapy might affect healthy human cells remains to be studied. Ligands to NCRs may be expressed primarily as a consequence of cellular stress, activation, viral infection, or tumor transformation [32], [33], therefore not by healthy cells; yet immature DC were reported to express ligands to NKp30 [34].

We demonstrate the therapeutic potential of this approach in two *in vivo* models of human prostate cancer; PC3/*Luc* and DU145 cell lines. We show that in both models a significant inhibition of tumor growth was achieved when animals were treated with NKp30-Ig fusion protein. Moreover, in 50% of the mice injected with either PC3/*Luc* or DU145 cell lines, treatment resulted in complete growth arrest of the tumor with no relapse. The emergence of ligand-negative tumor cells, following therapy-mediated selective pressure is a frequent phenomenon in tumor therapy. This could account for the unresponsiveness to treatment observed in fraction of the mice. We harvested tumor cells from two mice with progressive tumor growth: one treated with NKp30-Ig-and one treated with NKp46D2-Ig. Staining of the tumor cells with NKp30-Ig and NKp46D2-Ig revealed down-regulation of the ligand specific for the treatment (data not shown). Thus, tumor-escape variants could emerge following NCR-Ig treatment but more mice should be studied to accurately define this phenomenon.

The *in vivo* results demonstrated here indicate a potential novel therapeutic approach. Several ways should be considered in order to improve its effect. Importantly, since NKp30-Ig is not efficiently internalized upon binding to tumor cells (data not shown), its coupling to toxins is not likely to increase its anti-tumor abilities. However, alternative strategies may be considered, including arming the NKp30-Ig with radionuclides, attaching NKp30-Ig to the surface of liposomes for selective tumor targeting of chemotherapy toxins or even DNA for gene therapy, or using NKp30 as the tumor-specific epitope of a bi-specific antibody [20]. In addition, combined treatment with both NKp30-Ig and conventional chemotherapy should be tested. This strategy is of particular interest since clinical studies of antibody-based immunotherapy have clearly shown that in most cases, the best response is obtained when antibody treatment is combined with standard chemotherapy. For example, administration of Rituximad together with chemotherapy increased the response rates from 50% to 95% [35], [36]. Similar synergistic effects with no increase in toxicity were also documented for other anti-tumor antibodies, such as Herceptin and Erbitux, [37], [38]. The exact molecular events by which antibody-based immunotherapies exert their therapeutic effect are not always entirely understood and it is believed that multiple mechanisms are involved. However, both *in vitro* and *in vivo* studies indicate that antibody-dependent-cell-mediated cytotoxicity (ADCC) is the predominant mode by which an anti-tumor response is achieved [27]. The most conclusive evidence for the importance of an ADCC response in antibody-based immunotherapies comes from *in vivo* studies of knockout mice in which the activating Fc receptors were impaired [26], [39]. These studies clearly demonstrated that the therapeutic potency of two clinically effective mAbs, Herceptin and Rituxan, greatly depends on the expression of activating Fc receptors on immune cells. In agreement, mice deficient in the FcγRIIB inhibitory Fc receptor showed an enhanced ADCC response [26], [39]. Furthermore, monocytes and macrophages were suggested to function as the dominant effector cells in the antibody-mediated protection *in vivo* [26]. Our *in vitro* data suggest that NKp30-Ig may also mediate its therapeutic effect via a similar mechanism enhancing a macrophage-dependent ADCC response. The fact that the binding of NKp30-Ig to tumor cells *in vitro* is stable and does not induce internalization of its unknown ligand (data not shown) may increase the efficiency of an ADCC response.

While macrophages-mediated ADCC response may be an important way by which NKp30-Ig suppresses tumor growth *in vivo*, other additional mechanisms may also contribute to this effect. In this regard it should be noted that the inability of NKp30-Ig to produce any detectable changes in the cell cycle of PC3/*Luc* and DU145 cell lines *in vitro* may not necessarily be the case in the more complex environment *in vivo*. The absence of basic information regarding the nature of the tumor molecules that are recognized as ligands by the NCRs complicates this issue and prevents its direct examination. However, the abundant expression of potential NCRs ligands on clinically derived tumor samples suggests that *in vivo* these ligands are maintained, despite the probable selective pressure exerted by NK cells. Thus, it is possible to speculate that the NCRs ligands may be vital for the survival and progression of transformed cells. Binding of the Ig fusion proteins to these ligands may therefore contribute to tumor growth arrest by directly interfering with the activation of these molecules, and consequently suppress the tumorigenic process *in vivo*.

While both NKp30-Ig and NKp46D2-Ig showed specific and similar levels of binding to PC3/*Luc* and DU145 cell lines *in vitro,* treatment of mice with NKp46D2-Ig did not induce any therapeutic effect and no inhibition of tumor growth was observed in either the PC3/*Luc* or the DU145 xenografts. This observation may be explained by the relatively short half-life of the NKp46D2-Ig fusion protein.

Prostate cancer is one of the most prevalent cancers in males. More then 230,000 persons are diagnosed every year in the US. The progression of the disease includes a transition from an androgen-dependent stage, in which the cancer is organ-confined and still curable, to an androgen-independent stage, in which the tumor metastasizes to other organs and in many cases leads to death [22]. An accurate diagnosis of the disease stage is therefore of great importance and has direct implications on the treatment strategy. The early diagnosis usually relies upon the symptoms of the patient, the digital rectal examination (DRE) and the prostate specific antigen (PSA) level [40]. Unfortunately, none of these tests are accurate and reliable enough to clearly determine the stage of the disease and in order to gain a conclusive diagnosis a TRUS guided biopsy is required [41]. However, despite the fact that the biopsies are repetitively taken (at least 6 times from each patient), there is always the problematic risk of missing those tissues in which the most advanced stage of the tumor has been established. It is therefore critical to develop additional tests that would provide supplementary information and allow consistent distinguish between idle and aggressive prostate cancer. The ability of NKp30-Ig to effectively target prostate cancer *in vivo* may therefore be further applied as a diagnostic tool used as a marker in magnetic resonance spectroscopy (MRS) test. Previous attempts to exploit the MRS technique for prostate cancer diagnosis were proved fruitful [42]. Since NKp30-Ig selectively binds prostate adenocarcinoma, but not BPH, we hypothesize that it may provide additional valuable information that would help to distinguish between these two common pathological conditions.
