# Correction: Harnessing Soluble NK Cell Killer Receptors for the Generation of Novel Cancer Immune Therapy

**DOI:** 10.1371/annotation/eaf9794a-9325-4977-952d-51285c3f6c6a

**Published:** 2008-05-19

**Authors:** Tal I. Arnon, Gal Markel, Ahuva Bar-Ilan, Jacob Hanna, Eyal Fima, Fabrice Benchetrit, Ruth Galili, Adelheid Cerwenka, Daniel Benharroch, Netta Sion-Vardy, Angel Porgador, Ofer Mandelboim

The funding information for this paper is missing. It should read:

This work was supported by the Israel Science Foundation (O.M.), the Binational Science Foundation (O.M.), the AICR (O.M), the ICRF (O.M.), the European Commission (LSHC-CT-2002-518178 and MRTN-CT-2005 to O.M.), the Israel Ministry of Health (A.P.), the Cooperation Program in Cancer Research of the Deutsches Krebsforschungszentrum (DKFZ), and Israeli's Ministry of Science and Technology (MOST) (A.P.).

