# Correction: Harnessing Soluble NK Cell Killer Receptors for the Generation of Novel Cancer Immune Therapy

**DOI:** 10.1371/journal.pone.0128052

**Published:** 2015-05-04

**Authors:** Tal I. Arnon, Gal Markel, Ahuva Bar-Ilan, Jacob Hanna, Eyal Fima, Fabrice Benchetrit, Ruth Galili, Adelheid Cerwenka, Daniel Benharroch, Netta Sion-Vardy, Angel Porgador, Ofer Mandelboim

There are errors in Fig 4, “Summary of fusion protein treatment”. Fig 4a contains the wrong set of images for PBS (control) injected mice. Please see the corrected Fig 4 here.

**Fig 4:**
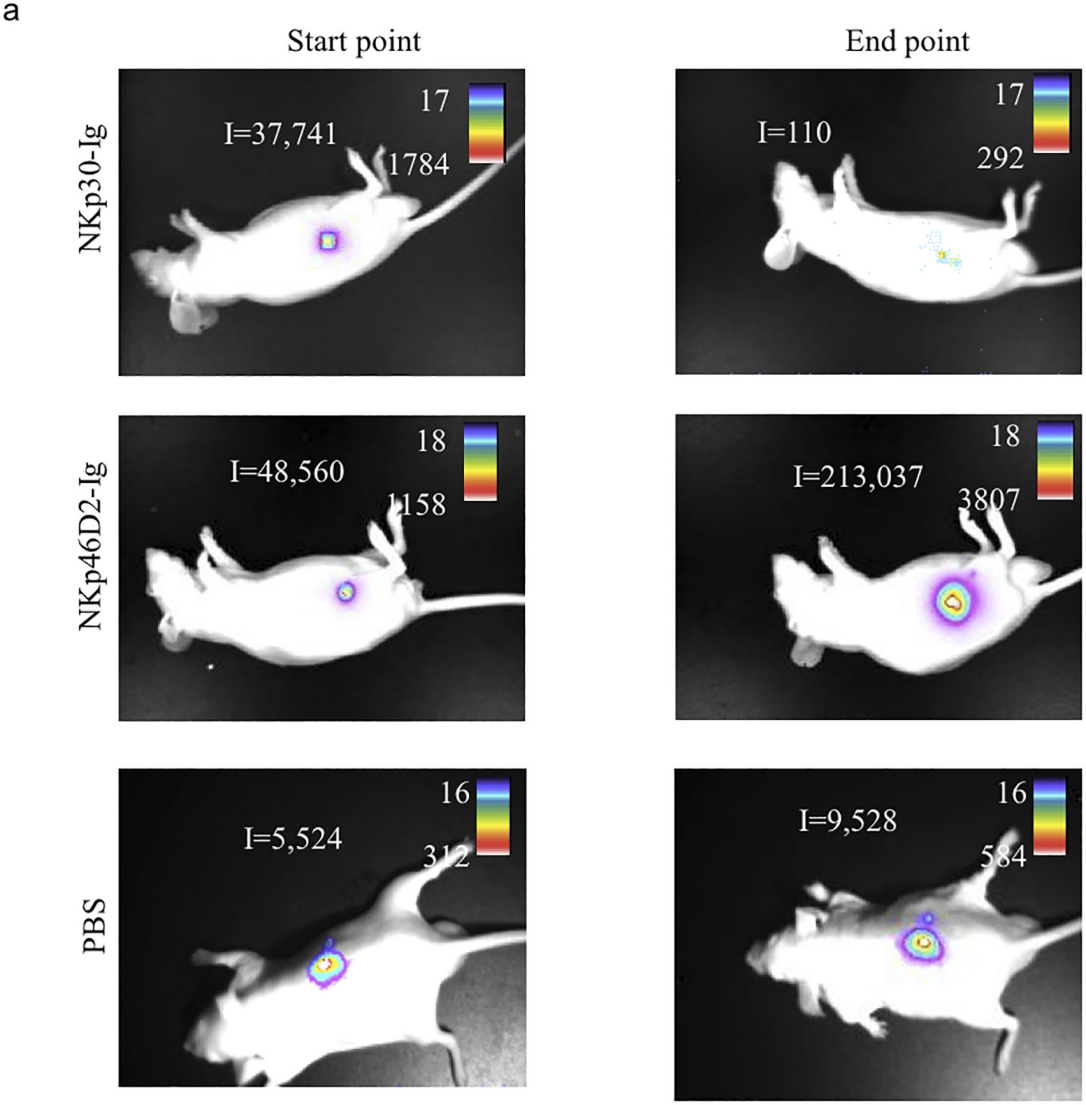
Summary of fusion protein treatment. (a) Visualization of tumor progression and distribution in vivo. The figure shows an image visualization of one representative animal of each treatment. The scale on the right of each figure describes the color map of the photon count. The integrated light emission (‘I’) is indicated in the left of each photo. (b) Summary of treatment effect. Table describes the overall effect of treatments, as shown in details in Fig 3.
